# The association between temperature, rainfall and humidity with common climate-sensitive infectious diseases in Bangladesh

**DOI:** 10.1371/journal.pone.0199579

**Published:** 2018-06-21

**Authors:** Fazle Rabbi Chowdhury, Quazi Shihab Uddin Ibrahim, Md. Shafiqul Bari, M. M. Jahangir Alam, Susanna J. Dunachie, Alfonso J. Rodriguez-Morales, Md. Ismail Patwary

**Affiliations:** 1 Department of Medicine, Bangabandhu Sheikh Mujib Medical University (BSMMU), Dhaka, Bangladesh; 2 Centre for Tropical Medicine and Global Health (CTMGH), University of Oxford, Oxford, United Kingdom; 3 Mahidol-Oxford Tropical Medicine Research Unit (MORU), Bangkok, Thailand; 4 Peter Medawar Building for Pathogen Research, University of Oxford, Oxford, United Kingdom; 5 Department of Medicine, Sylhet M.A.G. Osmani Medical College, Sylhet, Bangladesh; 6 Research Group and Incubator Public Health and Infection, Faculty of Health Sciences, Universidad Tecnologica de Pereira, Pereira, Risaralda, Colombia; University of West London, UNITED KINGDOM

## Abstract

Bangladesh is one of the world’s most vulnerable countries for climate change. This observational study examined the association of temperature, humidity and rainfall with six common climate-sensitive infectious diseases in adults (malaria, diarrheal disease, enteric fever, encephalitis, pneumonia and bacterial meningitis) in northeastern Bangladesh. Subjects admitted to the adult medicine ward of a tertiary referral hospital in Sylhet, Bangladesh from 2008 to 2012 with a diagnosis of one of the six chosen climate-sensitive infectious diseases were enrolled in the study. Climate-related data were collected from the Bangladesh Meteorological Institute. Disease incidence was then analyzed against mean temperature, humidity and average rainfall for the Sylhet region. Statistical significance was determined using Mann-Whitney test, Chi-square test and ANOVA testing. 5033 patients were enrolled (58% male, 42% female, ratio 1.3:1). All six diseases showed highly significant (p = 0.01) rises in incidence between the study years 2008 (540 cases) and 2012 (1330 cases), compared with no significant rise in overall all-cause hospital admissions in the same period (p = 0.19). The highest number of malaria (135), diarrhea (266) and pneumonia (371) cases occurred during the rainy season. On the other hand, the maximum number of enteric fever (408), encephalitis (183) and meningitis (151) cases occurred during autumn, which follows the rainy season. A positive (P = 0.01) correlation was observed between increased temperature and the incidence of malaria, enteric fever and diarrhea, and a negative correlation with encephalitis, meningitis and pneumonia. Higher humidity correlated (P = 0.01) with a higher number of cases of malaria and diarrhea, but inversely correlated with meningitis and encephalitis. Higher incidences of encephalitis and meningitis occurred while there was low rainfall. Incidences of diarrhea, malaria and enteric fever, increased with rainfall, and then gradually decreased. The findings support a relationship between weather patterns and disease incidence, and provide essential baseline data for future large prospective studies.

## Introduction

Global warming is not a myth, rather a reality [1, 2], and the impact of climate change is multidimensional on health. The World Health Organization (WHO) recently reported an estimated 12.6 million deaths each year due to unhealthy environments, particularly climate change and pollution [3]. The report also identifies diarrheal diseases, respiratory infections and malaria in the top ten causes of environment related deaths [3]. Climate change influences the emergence and re-emergence of many infectious diseases [1, 2, 4, 5]. Dengue fever, tick-borne diseases, diarrheal disease, enteric fever, viral encephalitis, respiratory tract infections, and meningitis are much increased in recent times [1–4, 6]. In addition, emerging and reemerging diseases such as chikungunya and Zika, have also been linked to climate change influences, and have been proposed as partially responsible for autochnous transmission in places not traditionally endemic for such diseases [7, 8].

Analysis of global temperature data led the Intergovernmental Panel for Climate Change (IPCC) to the conclusion that the average global temperature over land and ocean surfaces has risen by 0.85°C in the period from 1880 to 2012 [1]. They predicted that global surface temperature will increase by 1.5°C compared to the year 1850 by the end of the 21st century (2081–2100) [1]. Climate change is predicted to directly influence zoonotic infectious disease transmission by changing the geographic range of a vector [9]. Altered climatic conditions may increase vector biting rate and the reproduction rate of the vector and shorten the pathogen incubation period [9–12]. Climate-related increases in sea surface temperature and sea levels can lead to higher incidence of waterborne infectious diseases [9, 13, 14].

As a low-income country, Bangladesh itself plays very little role in the process of global warming, but becomes one of the most seriously affected victims of climate change. Bangladesh is the biggest delta and contains the second largest river basin in the world [15]. The majority of the land is low and flat, and only 10% lies over one meter above the mean sea surface [15]. Because of monsoon weather and the presence of the Bay of Bengal in the south, extreme weather events like flood and cyclone are common [15, 16]. This unique geographic and topographic location makes it reportedly the most vulnerable country to climate change effects [16]. Lack of resilience and adaptive capacity, dense population and poverty make the situation worse [16]. Unfortunately, very few studies on the relationship between various environmental variables and trends of infectious disease incidence have been performed so far in Bangladesh, although there are reports of some infections increasing sporadically in different regions of the country [17–19]. Climate change and health related studies are so far mainly reported from developed countries, but studies from vulnerable countries are still meagre [20, 21]. Furthermore, published studies typically only focus on a single disease.

This study examined six infectious diseases based on clinical syndromes and laboratory support (malaria, enteric fever, encephalitis, diarrheal disease, pneumonia and meningitis) to offer a broader scope on the trend of these infectious diseases and their possible relation to climate change in Bangladesh. We chose these six diseases based on the reports of IPCC (2014) and WHO (2016) where they were listed as climate-sensitive infectious diseases important for Asia [1–3]. The main objective of the study was to see the burden of the six climate sensitive diseases over five years and to analyze the possible relationship of them with common climatic variables. The findings will be of interest to public health experts and policy makers to stimulate effective measures to combat infectious diseases and related epidemics in Bangladesh and in other vulnerable countries.

## Materials and methods

This was an observational exploratory study done at Sylhet M.A.G. Osmani Medical College Hospital (SOMCH), Sylhet, Bangladesh. This is a 1200 bed tertiary referral centre covering the Sylhet divison in Northeast Bangladesh, an area of approximately 12,298 km^2^ with a population of around 10 million. All case files of the adult medicine ward from 2008 to 2012 were enrolled from the hospital archive. The diagnosis of the six studied diseases were confirmed based on the combination of clinical and relevant laboratory diagnosis. Incomplete files, patients discharged on risk bonds, patients who died within 24 hours (due to insufficient time to reach a diagnosis) and absconded patients were excluded from the study.

### Case definitions

#### Enteric fever

High-grade fever for more than 7 days with abdominal symptoms (loose motion/ abdominal pain/ constipation/ vomiting) with or without blood culture positive for *Salmonella typhae* and *paratyphae*.

#### Diarrheal diseases

Diarrhea is defined as the passage of three or more loose or liquid stools per day (or more frequent passage than is normal for the individual) with signs of dehydration, with or without fever and with or without positive stool microscopy and culture.

#### Bacterial meningitis

High Fever with or without unconsciousness with signs of meningeal irritation and positive CSF diagnostic of bacterial meningitis (raised white cell count, low glucose with or without culture of an appropriate bacterial pathogen.

#### Encephalitis

High fever with altered consciousness and positive CSF findings diagnostic of Encephalitis (raised lymphocyte cell count, mild to moderate elevated protein with normal glucose) with or without signs of meningeal irritation.

#### Pneumonia

High fever with cough, positive respiratory signs and consolidation on chest- X ray with or without positive sputum findings.

#### Malaria

High fever with chills and rigors with or without unconsciousness with or without blood film positive for malaria species, and response to antimalarial treatment within 48 hours.

#### Climate data

The mean temperature in degree Celsius, mean humidity in percentage and average rainfall in millimeters of the respective years (2008–2012) were chosen as climatic variables. These data were collected from the Bangladesh Meteorological Office, Agargaon, Dhaka. Only the information of the Sylhet weather station was included in the analysis. The data were available month-wise. Seasons were divided into four equal groups of three months duration; summer (March to May), rainy season (June to August), autumn (September to November) and winter (December to February).

#### Statistical analysis

The results were expressed as mean ± standard deviation and the level of significance was expressed as P values unless otherwise stated. Between the years 2008 to 2012, the mean temperature was 25.0°C, average humidity 76.5% and average rainfall was 275mm in Sylhet region. Based on this mean data, the average temperature was subdivided into two groups; <25.0°C and ≥25.0°C, humidity into three groups; <76.5%, 76.5–77.5% and >77.5%, and rainfall into three groups; <275 mm, 275–375 mm and >375 mm for data analysis. The Mann-Whitney test, Chi-square test, student t test and ANOVA test were applied to obtain the level of significance. Statistical analysis was performed using IBM SPSS Statistics 22 for Windows, with results displayed using GraphPad Prism 6.

#### Ethical approval

A statistician anonymized all data before analysis. The institutional ethical review committee of Sylhet M.A.G. Osmani Medical College approved this study prior commencement and the requirement for informed consent was waived.

## Results

**Fig 1:**
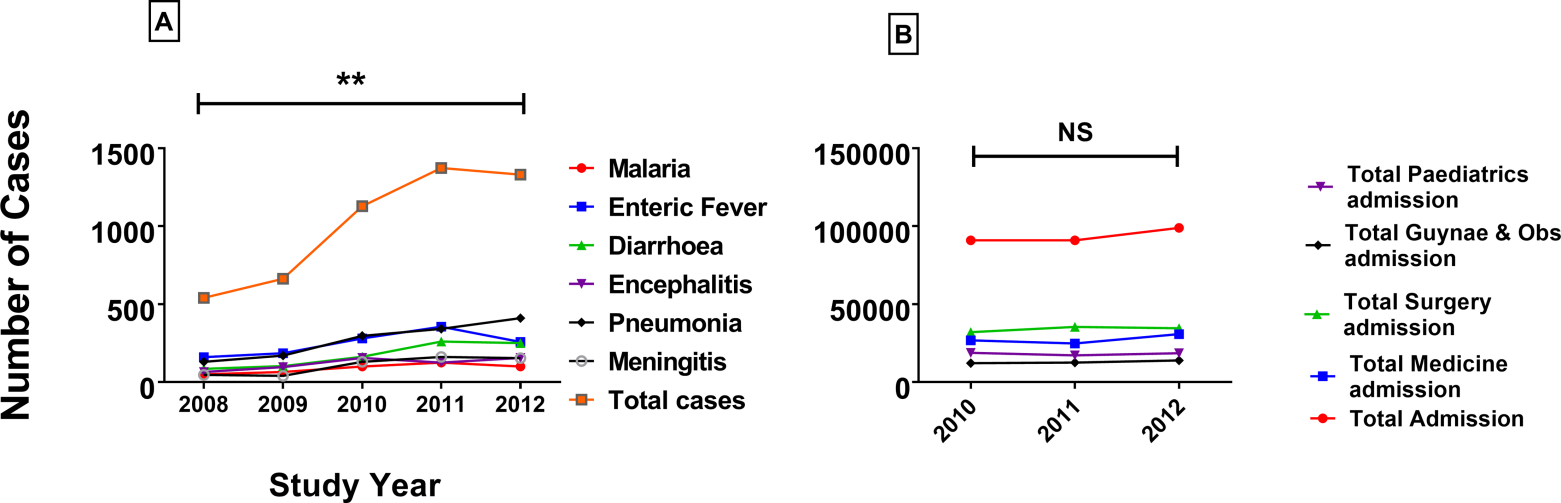
**A. Year-wise distribution of cases from the year 2008 to 2012.** Significant increase in the total number of cases was seen in 2012 compared to 2008. Mann-Whitney U test was applied for level of significance (p = 0.01). **B. All hospital admissions during 2010 to 12.** There were no significant differences in terms of total admissions between 2010 and 2012 in the study hospital. P value (0.191) expressed through Mann-Whitney U test.

5034 patients were enrolled into the study, with 2910 males (58%) and 2124 females (42%), ratio 1.3: 1. Pneumonia occurred significantly more frequently in males (72% vs. 28%, chi square P<0.001) whereas Encephalitis occurred more frequently in females in females (53% vs. 47%, chi square P<0.001) (Table 1). The highest percentage (27%; 1373) of disease over the five years studied occurred in the 4^th^ year (2011). There was a trend towards increasing number of cases of the six infectious diseases studied over the five years (Fig 1A) and there is a significant difference (P = 0.01) between the total number of cases in 2008 (540) and 2012 (1330). All six infectious diseases individually increased in number over the five years, especially pneumonia which rose from 131 cases in 2008 to 411 cases in 2012. This increase does not appear to be due to increased numbers of patients using the hospital overall. In comparison, the number of admissions for all causes to the hospital and to the medicine department over three of the years of study from 2010 to 2012 (data unavailable for 2008 and 2009) did not change (Mann-Whitney U test p = 0.191, Fig 1B).

**Table 1:** Distribution of diseases according to sex (2008–2012).

Disease	Male	Female	Total
Malaria	228	215	443
Enteric Fever	718	523	1241
Diarrheal Diseases	452	411	863
Pneumonia	968	384	1352
Encephalitis	281	320	601
Bacterial Meningitis	263	271	534

### Disease and different seasons

**Fig 2:**
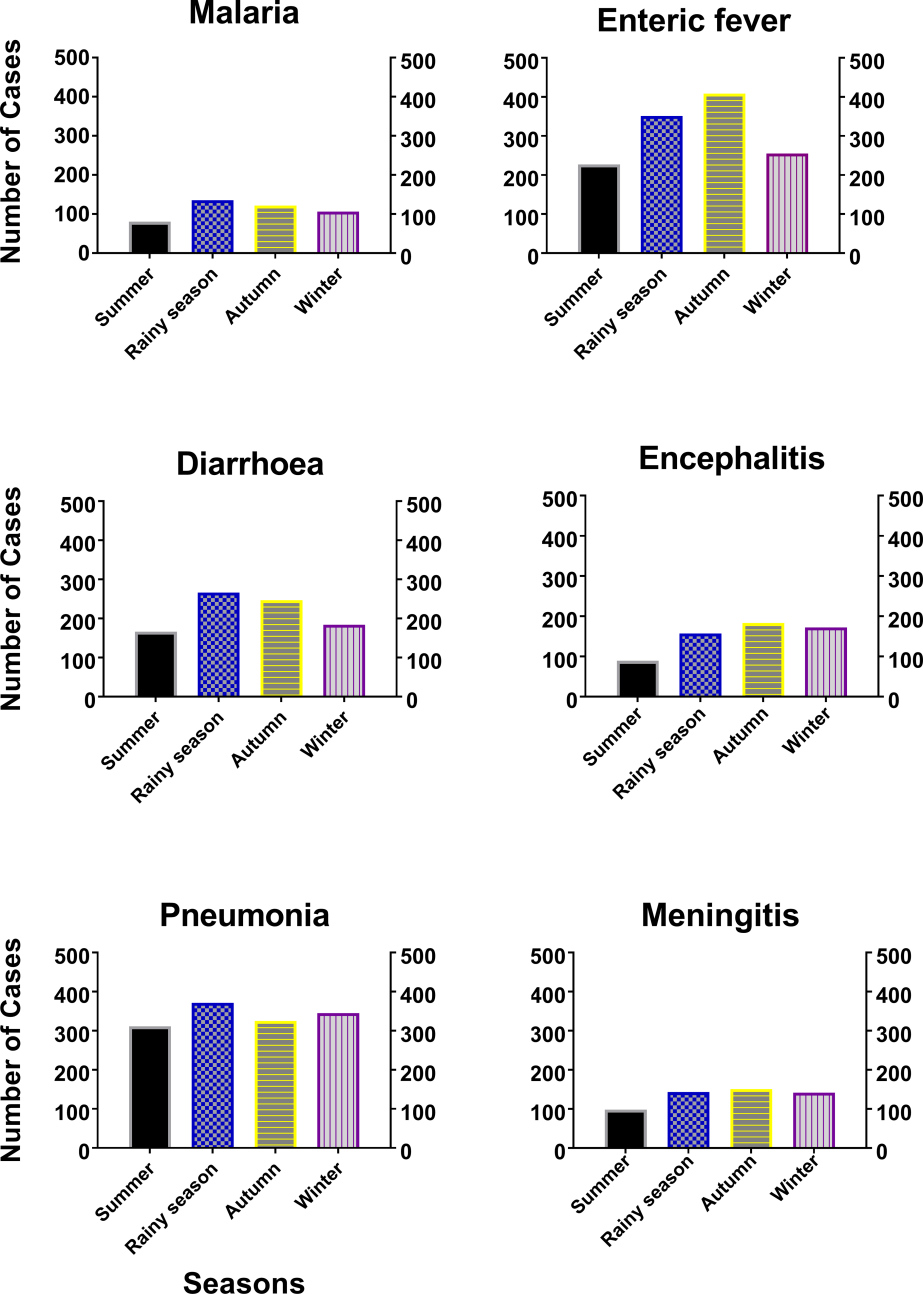
Association of disease with different seasons during the study period (2008–12). The highest number of malaria, diarrhea and pneumonia cases occurred in rainy season, whilst the maximum number of enteric fever, encephalitis and meningitis recorded during autumn. Student t test was applied to obtain the level of significance.

The highest number of cases occurred in autumn (1436; 29%) followed by rainy season (1423; 28%), winter (1204; 24%) and summer (971; 19%). Pneumonia (1352; 27%) was the most common disease of the six, and malaria (443; 9%) was the least prevalent disease. Individually, the highest number of malaria (135), diarrhea (266) and pneumonia (371) cases occurred in rainy season. On the other hand, the maximum number of enteric fever (408), encephalitis (183) and meningitis (151) cases occurred during autumn (Fig 2).

### Diseases and relationship with average temperature

**Fig 3:**
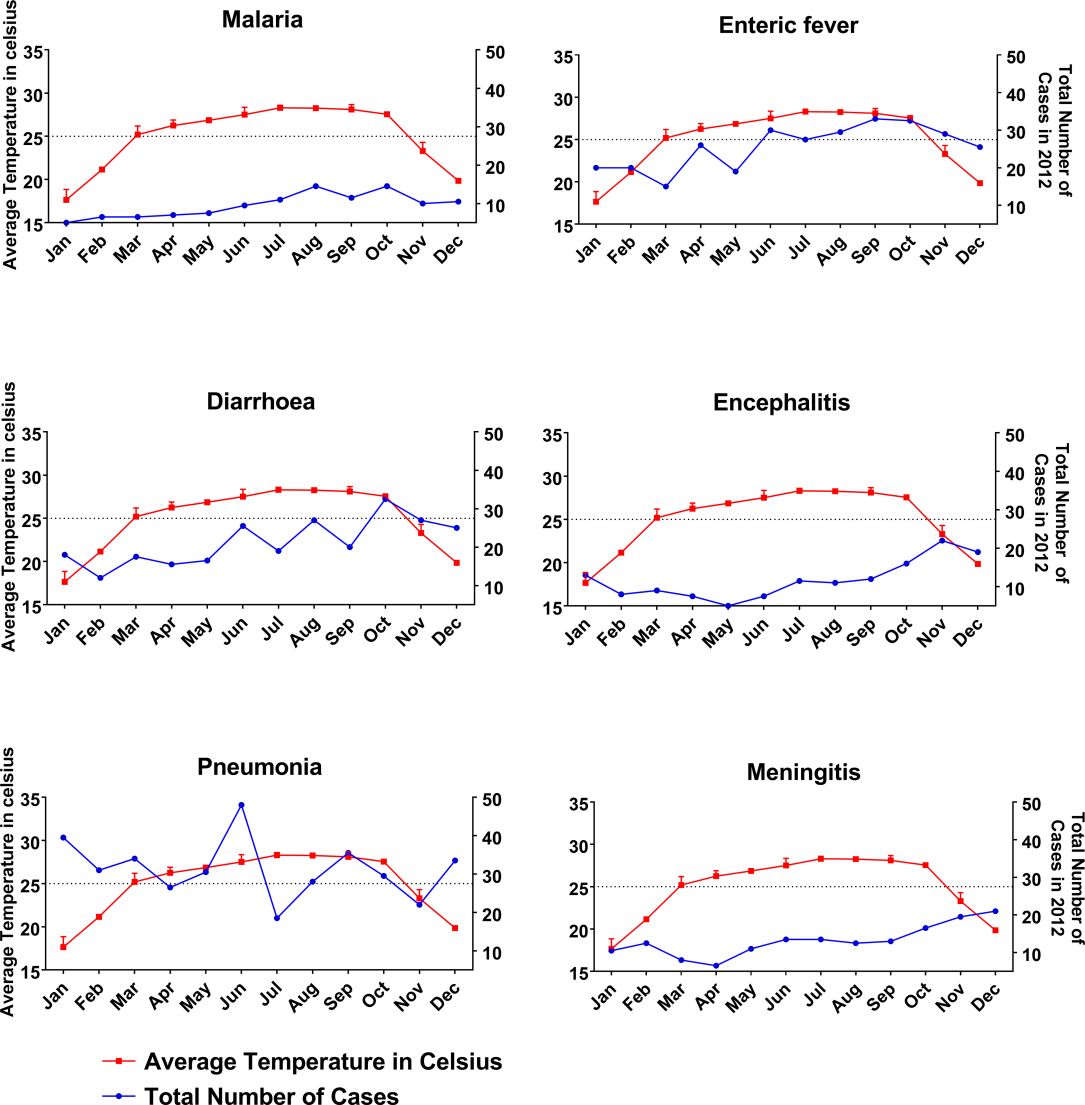
Association of disease with the average temperature for the study year 2012. An increased number of malaria, enteric fever and diarrhea cases was observed with increased temperature. On the other hand, the cases of encephalitis, meningitis and pneumonia were low. Two-way ANOVA test was applied to obtain the level of significance.

The mean temperature in our study region during 2008–12 was 25.0°C, and there was no significant difference in the number of cases occurring below or above that level (Table 2). In 2012, the average temperature was more than 25°C in eight out of twelve months (Fig 3). There was a significant positive correlation between increasing temperature and the incidence of malaria (p = 0.0001), and a trend for positive correlation between increasing temperature and the incidence of diarrhea (p = 0.081). There was a significant negative correlation between increasing temperature and incidence of encephalitis (p = 0.001), meningitis (p = 0.001) and pneumonia (p = 0.017).

**Table 2:** Association of disease with the temperature of five years (2008–2012).

Climatic variable	Malaria n (%)	Enteric Fever n (%)	Diarrheal Diseases n (%)	Encephalitis n (%)	Pneumonia n (%)	Bacterial Meningitis n (%)	Total n (%)
Temperature							
<25^o^ C	217 (49.0)	604 (48.7)	439 (50.9)	319 (53.1)	713 (52.7)	241 (45.1)	2533 (50.3)
≥25^o^ C	226 (51.0)	637 (51.3)	424 (49.1)	282 (46.9)	639 (47.3)	293 (54.9)	2501 (49.7)
**Total n (%)**	443 (100.0)	1241 (100.0)	863 (100.0)	601 (100.0)	1352 (100.0)	534 (100.0)	5034 (100.0)

### Diseases and relationship with average humidity

**Fig 4:**
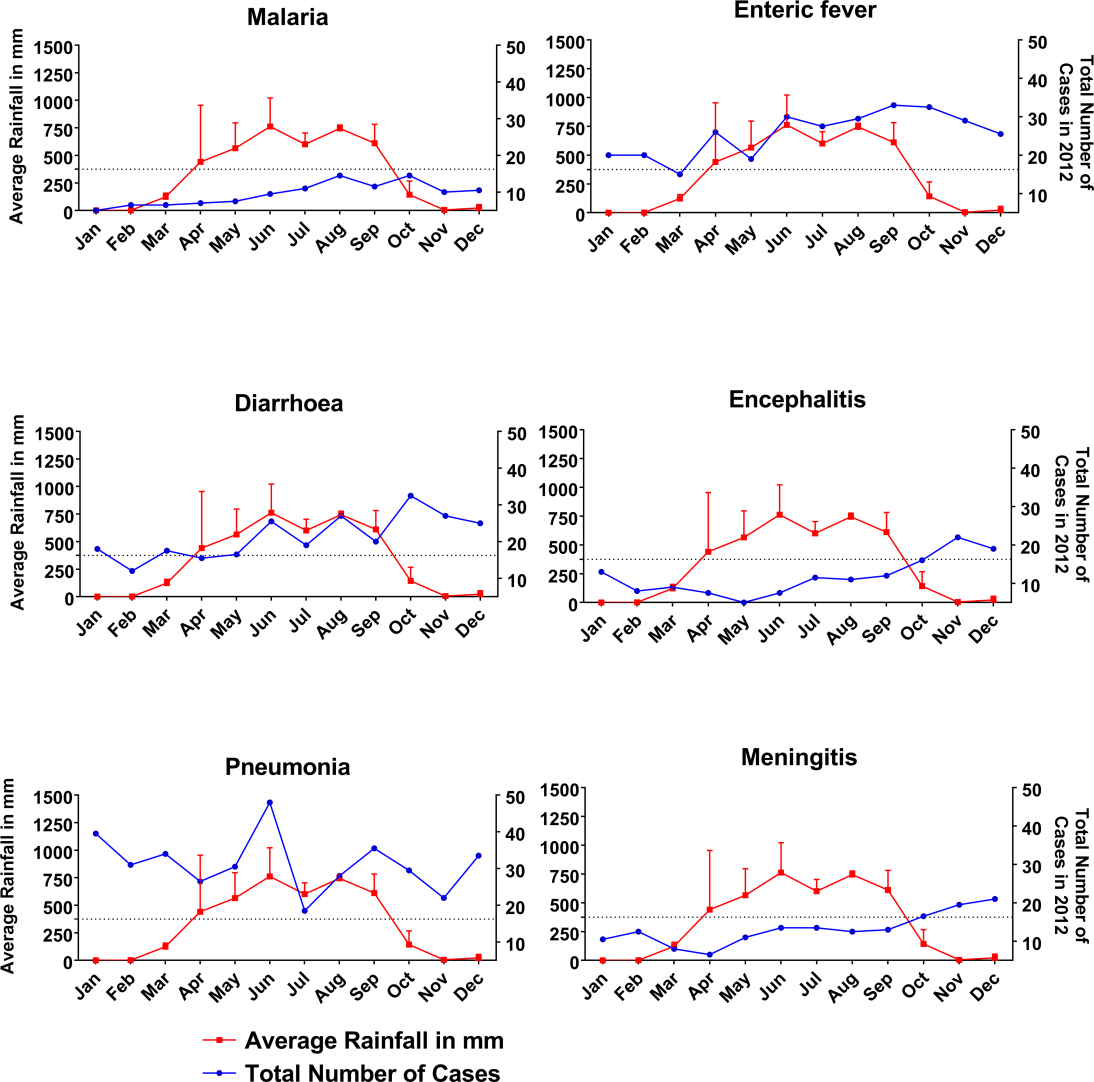
Association of disease with the average humidity for the study year 2012. Higher humidity was correlated with a higher number of cases of malaria, enteric fever and diarrhea, but inversely correlated with meningitis, encephalitis and pneumonia. Two-way ANOVA test was applied to obtain the level of significance.

Table 3 shows the association of the six diseases with the average humidity of the studied period of 2008 to 2012. We analysed the incidence of each disease in each of three groups of humidity recordings by month: less than 76.5%, 76.5–77.5% and more than 77.5% based on the mean humidity of the study site. Less than 76.5 percent humidity was associated with the highest percentage (2458; 49%; p = <0.01) of all the six studied diseases (Table 3). Average humidity of 76.5–77.5% and > 77.5% was associated with 27% (1373) and 24% (1203) disease respectively. However, in 2012, the average humidity was ≥ 76.5% in nine out of twelve months (Fig 4). Higher humidity was correlated with a higher number of cases of malaria (p = 0.0001), enteric fever (p = 0.0001) and diarrhea (p = 0.0001), but inversely correlated with meningitis (p = 0.0001), encephalitis (p = 0.0001) and pneumonia (p = 0.0001).

**Table 3:** Association of disease with the humidity of five years (2008–2012).

Climatic variable	Malaria n (%)	Enteric Fever n (%)	Diarrheal Diseases n (%)	Encephalitis n (%)	Pneumonia n (%)	Bacterial Meningitis n (%)	Total n (%)
Humidity							
<76.5%	201 (45.4)	539 (43.4)	413 (47.9)	312 (51.9)	708 (52.4)	285 (53.4)	2458 (48.8)
76.5% -77.5%	126 (28.4)	356 (28.7)	261 (30.2)	126 (21.0)	342 (25.3)	162 (30.3)	1373 (27.3)
>77.5%	116 (26.2)	346 (27.9)	189 (21.9)	163 (27.1)	302 (22.3)	87 (16.3)	1203 (23.9)
**Total n (%)**	443 (100.0)	1241 (100.0)	863 (100.0)	601 (100.0)	1352 (100.0)	534 (100.0)	5034 (100.0)

### Diseases and relationship with average rainfall

**Fig 5:**
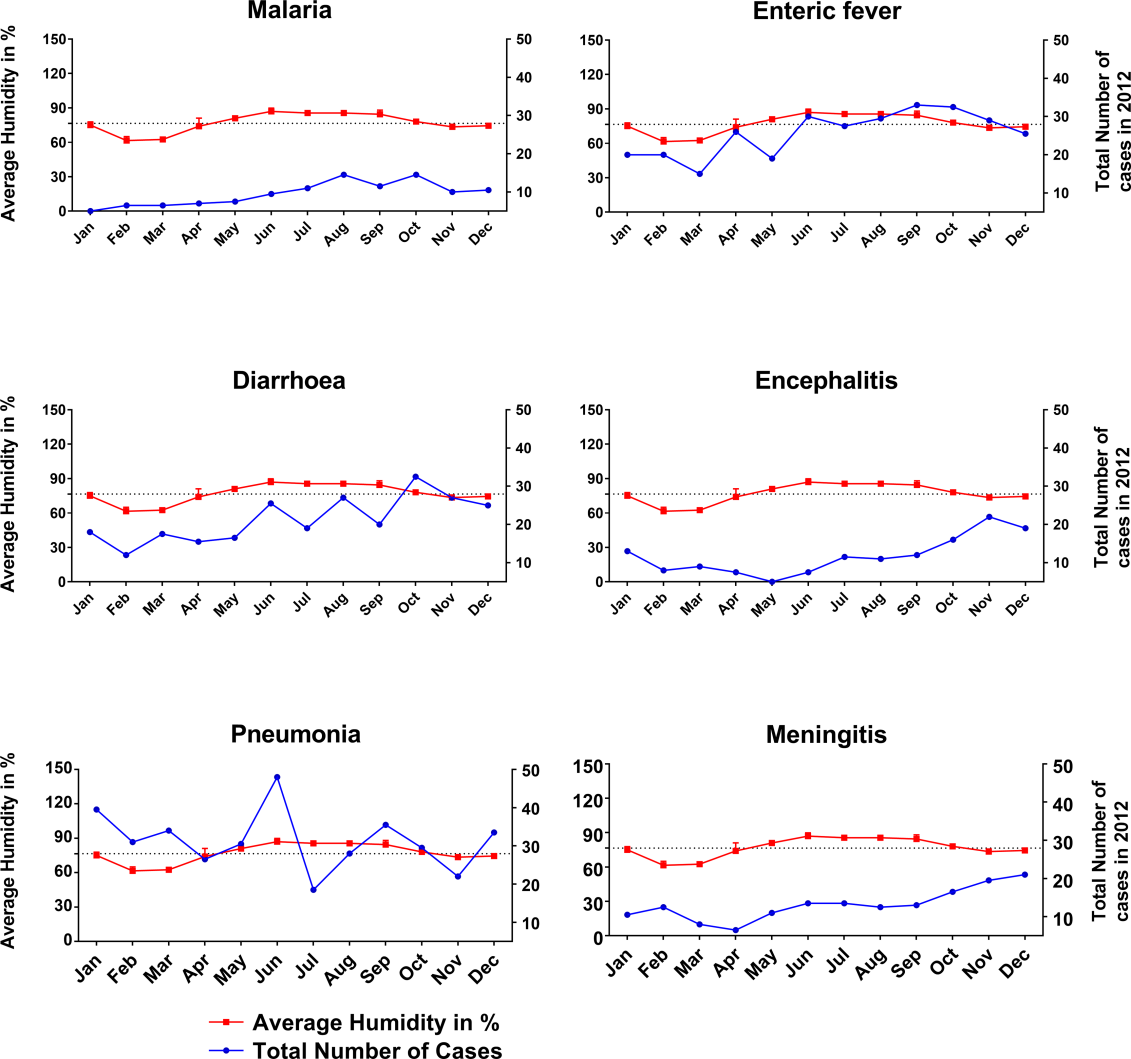
Association of disease with the average rainfall for the study year 2012. Higher incidences of encephalitis and meningitis occurred while there was low rainfall. Incidences of diarrhea, malaria, pneumonia and enteric fever increased with rainfall. Two-way ANOVA test was applied to obtain the level of significance.

The average rainfall by month was divided into three groups, less than 275 mm, 275–375 mm and more than 375 mm in Table 4. The highest percentage (2458; 48.8%) of all the six diseases studied were related with less than 275 mm of average rainfall. 45% (201) malaria, 43% (539) enteric fever, 48% (413) diarrhea, 52% (312) encephalitis, 52% (708) pneumonia and 53% (285) meningitis fell under this category (chi-square p = 0.010). Average rainfall of 275–375 mm and > 375mm was associated with 24% (1203) and 27% (1373) of the total disease burden respectively. Analyzing the data of 2012, in six out of twelve months, there were ≤375 mm average rainfall (Fig 5). Higher incidences of encephalitis (p = 0.001) and meningitis (p = 0.001) happened while there was low rainfall. Incidences of diarrhea (p = 0.002), malaria (p = 0.001), pneumonia (p = 0.002) and enteric fever (p = 0.002) increased with rainfall, and then gradually decreased.

**Table 4:** Association of disease with the rainfall of study years (2008–2012).

Climatic variable	Malaria n (%)	Enteric Fever n (%)	Diarrheal Diseases n (%)	Encephalitis n (%)	Pneumonia n (%)	Bacterial Meningitis n (%)	Total n (%)
Rainfall							
<275 mm	201 (45.4)	539 (43.4)	413 (47.9)	312 (51.9)	708 (52.4)	285 (53.4)	2458 (48.8)
275 mm—375 mm	116 (26.2)	346 (27.9)	189 (21.9)	163 (27.1)	302 (22.3)	87 (16.3)	1203 (23.9)
>375 mm	126 (28.4)	356 (28.7)	261 (30.2)	126 (21.0)	342 (25.3)	162 (30.3)	1373 (27.3)
**Total n (%)**	443 (100.0)	1241 (100.0)	863 (100.0)	601 (100.0)	1352 (100.0)	534 (100.0)	5034 (100.0)

## Discussion

Previous studies in Bangladesh have either focused on a single disease or relied on people’s perception of climate change and infectious disease. To our knowledge, this study is the first observational study which specifically focuses on individual infectious diseases and explores the relationship between each disease and three common weather variables. The male to female ratio in this study was 1.3: 1. Although there are no published data, it is generally believed that more males come into hospital in Bangladesh because traditionally females wait at home with their disease until complications develop [22, 23]. It is likely that women get less opportunity to come into hospital and have less access to medical care.

There was no significant difference by gender in the frequency of each individual disease except pneumonia and encephalitis, which significantly affected a greater proportion of the male and female population respectively. The increase in pneumonia cases seen in males may be because men have more occupational exposure to conditions that increase the chance of receiving a diagnosis of pneumonia such as dust, fumes, smoking etc. All six diseases showed a significant (P = 0.01) rise in incidence between the study years 2008 and 2012. The number of cases more than doubled for all diseases except enteric fever, which also showed significant increases. In contrast, during this same period the total number of admissions to the medical ward and to in the hospital showed no significant rise. We propose that the rise in frequency of the studied diseases could be explained by the influence of weather changes. It is important to note that during this period no epidemics of the studied diseases happened in the Sylhet region. The total impression is consistent with previous reports. In a review of the literature, the incidence of pyogenic meningitis, encephalitis and dengue was predicted to be greatly influenced and increased by global warming in the coming years [24]. W.H.O reported dengue, viral encephalitis, diarrheal disease, enteric fever, pneumonia and meningitis as most sensitive to climate factors, and predicted a huge rise of cases in tropical countries [25]. *Climate Change Cell*, Bangladesh reported that, from 1984 to 1993 there were 301,651cases of malaria in Bangladesh, but from 1994 to 2003 it increased to 507,485 (68% increased incidence) [26]. Although after the introduction of artemisinin treatment and government and other partner organization lead massive drive for malaria elimination, the cases decreased to 1.4/1000 population in Bangladesh [27]. The same report revealed an increasing trend for diarrheal diseases, Kala-azar and skin diseases in three districts (drought-prone Rajshahi, flood-prone Manikganj and salinity-dense Satkhira) of Bangladesh between 1999 to 2005 [26]. The report also described a positive correlation between rainfall and diarrheal and skin disease in Rajshahi and Satkhira, and a negative correlation of diarrheal disease with temperature [26]. Another focus group discussion (FGD) based study reported an increased number of diarrheal diseases, typhoid and skin problems after the cyclone Sidr and Aila in southern part (Barguna and Khulna) of the country [28].

We found the highest number of malaria (135), diarrhea (266) and pneumonia (371) cases occurred during the rainy season. The findings are consistent with other national and international studies. Highest cases of falciparum malaria were found in north-eastern India during the rainy season [29]. In the Chittagong hill tract districts of Bangladesh, where malaria is most endemic, the frequency of cases was highest in rainy season [30]. An increased incidence of malaria in north-west of India has been suggested through computational modelling [31]. Studies in Africa revealed mixed results, with the highest number of malaria cases during the rainy season in Mali, but most cases during autumn in Northern Ghana [32, 33].

According to the IPCC report, respiratory infections also follow a seasonal pattern [2]. In tropical settings, where most deaths due to pneumonia occur, the incidence of lower respiratory tract illness in children is generally increased during rainy season and it supports our findings [34]. A Thai study of viral pneumonia reported the highest number of cases in rainy season [35], in line with our findings. This pattern of increased pneumonia cases during rainy season in tropical countries contrasts with the well described increase in pneumonia seen during colder months in temperate climates [36, 37]. For diarrheal disease, our findings are supported by previous reports of increased incidence during the rainy season in Taiwan (24) and Bangladesh (25).

We found a large number of enteric fever cases (759; 61%) occurred in rainy season and autumn. This agrees with earlier studies in both Dhaka, Bangladesh (26) where the highest number (45%) of enteric fever occurred during monsoon period [38],and a Cambodian study [39] but no relationship between incidence of enteric fever and season was seen in Kenya [40]. In our study, most meningitis (293; 55%) and encephalitis (355; 59%) occurred during autumn and winter. This finding is partly consistent with other studies done in Africa. Meningitis epidemics in West Africa occurred during the coolest season [41]. A recent time series analysis over 66 countries found that bacterial meningitis season peaks during the winter months, [42], similar to our findings. A major causative organism of meningitis (*Neisseria meningitides*) was found to be high and active during dry periods in the presence of dust and were then washed away with rainfall, so as the case frequency fell down [43, 44]. However, in our study, we found an almost equal number of meningitis cases in rainy season compared to other seasons. Regional findings of seasonality in terms of encephalitis are also supportive of our findings. Highest number of encephalitis cases in rainy period (August-September) were seen in Nepal [45]. In India, the incidence of Japanese encephalitis was also highest during August to November (rainy and early winter) with a peak in October [46]. Another study from China also reported similar findings [47].

The current rate of increase of the annual minimum temperature (by 0.1°C) is higher than that of the annual maximum temperature (by 0.09°C) in Bangladesh [48]. The annual average rainfall is increasing by 10.6 mm per decade outside the usual rainy period, while rainfall during the season is decreasing by 7.6 mm per decade [49, 50]. In our study, we found a positive correlation between the incidences of malaria, enteric fever and diarrhea with increasing temperature. These results are similar to other national and international findings. A previous study in Chittagong, Bangladesh showed increased malaria cases with increasing temperature [30]. Studies have shown a positive relationship between increased malaria and increasing temperature [51, 52]. Studies in South Asia and South America (Venezuela and Columbia) have documented the association between malaria outbreaks and the El Nino Southern Oscillation (ENSO) cycle [53]. A significant increase in malaria cases with increasing temperature was seen in Rwanda and Uganda [54, 55], but this was not seen in studies done in the African highland region [56, 57]. Increased temperature allows faster replication of mosquito populations [47, 58]. Higher temperatures also change human behavior, for example more outdoor activities may be undertaken which further increases the risk of exposure [47]. Diarrheal cases including dysentery have been found to be higher in high temperature in Bangladesh, Taiwan and China [59–61]. A study in Dhaka, Bangladesh showed an increase number of enteric fever cases with an increase temperature which supports our findings [38], as does a similar study from Southern Australia[62]. Furthermore, a strong linear association has been noted between temperature and notification of salmonellosis globally [53]. Temperature affects the transmission of food-borne disease in various ways. The temperature directly affects the rate of replication of bacterial and protozoan pathogens and the survival of enteroviruses in the environment [61, 63]. In addition, these variations may also have a significant impact on the environmental reservoirs of infection as well as human behavior [63]. Moreover, both salmonella and cholera bacteria, for example, proliferate more rapidly at higher temperatures; salmonella in animal gut and food, cholera in water [13, 53]. Our study shows an inverse relationship between encephalitis, meningitis and pneumonia with temperature. The pneumonia findings are consistent with a study done in China, but not with another study conducted in Spain [36, 64]. For encephalitis and meningitis our findings differ to other studies done in India, China and Niger where they found increased evidence of cases and vector during hot environment [47, 65, 66].Viral encephalitis cases in Sweden have reportedly increased in response to a succession of warmer winters over the past two decades [53]. This inverse relationship with temperature in our study merits further exploration.

In recent decades, more heatwaves have been reported in South Asia, which also bring a change in humidity [67]. Eighteen heatwaves were reported in India between 1980 and1998 [67]. Our study found a higher trend of malaria, enteric fever and diarrhea cases with higher humidity. The findings are further supported by studies done in Chittagong, Bangladesh and China with similar trend [30, 61]. Relative humidity influences biological and feeding behavior of mosquitoes [47]. At higher humidity, mosquitoes generally survive for longer and disperse further [47, 58]. Higher humidity also affects the rate of replication of bacterial and protozoan pathogens and their survival in the environment [63]. We did not find any influence of humidity on enteric fever and pneumonia, in line with other studies [34, 36]. The incidence of pneumonia, encephalitis and meningitis is inversely related to humidity in this study. In India an inverse association was seen between the number of Culex mosquitoes (the vector for Japanese encephalitis) and humidity [66], although in China, higher cases of encephalitis was seen with higher humidity [47]. These mixed findings may reflect differences in etiology of encephalitis and bacterial meningitis, as well as differences in case definitions between study sites. These could also explained by diagnostic limitations, and in some cases the meningoencephalitis syndrome may be caused by bacteria favoring dry and dusty weather.

Heavy rainfall can have a diverse range of effects on disease. For example, in tropical and subtropical regions with crowding and poverty, heavy rainfall and flooding may trigger behavioral changes such as increased contact between people and distribution of pathogens in floodwater, leading to outbreaks of diarrhea [58]. We found that the incidence of diarrhea, malaria, enteric fever and pneumonia increased with rainfall. For diarrhea, our findings are consistent with other national and international studies [59–61]. A positive relationship between increased rainfall and the incidence of enteric fever was also found in Dhaka, Bangladesh and southern Australia [38, 62]. One possible explanation is, heavy rainfall may affect the frequency and level of contamination of drinking water and hence the spread waterborne infections [13, 68]. Area with existing high burdens of infectious disease and poor sanitary infrastructure often experience increased rates of diarrheal diseases after heavy rainfall [58]. Our findings in terms of malaria are also similar to other national and regional studies [30, 31]. Rainfall plays an important role in the transmission of malaria, as mosquitoes need water to support the larval and pupal stages of development [31, 58]. A study also found higher deaths from acute lower respiratory tract illness during rainy season in a pediatric population [34]. In 2011–12, the highest incidences of encephalitis and meningitis occurred while there was very low rainfall. Our findings are supported by other reports in the case of meningitis [44, 65]. Nevertheless, researchers found the opposite for Japanese encephalitis, where the vector replicates and transmits more disease with high rainfall [45, 66].

We acknowledge a limitation of this study is the relatively short timeframe. Extending our study duration to ten years would have allowed greater power to detect differences, but we did not have the resources to do this. Another limitation is that due to the retrospective design of the study with recruitment from the hospital archive, we relied on the diagnosis in the patient notes, which in some instances may be wrong. We tried to overcome this limitation by setting case definitions for enrollment in the study and included only the cases who survived for more than 24 hours, where there was more clinical information to make an informed diagnosis. The death rate in the first 24 hours due to these infections (Bacterial meningitis-0.92%; encephalitis 2.32% and all-cause mortality 3.62%) during that period was low and unlikely to influence our results [69]. Due to limitations of funding and time, we conducted this study in only one hospital of northeastern region of Bangladesh. Further studies in other regions of Bangladesh are highly desirable to represent the situation across the country.

This study reported the influence of temperature, humidity and rainfall on six climate sensitive infectious diseases in the northeastern region of Bangladesh. Weather and climate extremes affect all sectors of the health, economy and development. The findings can be used as a baseline to launch a large cohort study throughout the country in future. This study is pivotal in giving direction to our public health experts, clinicians and other policy makers to update and change future strategies to combat the burden of climate-related health events in Bangladesh and other countries.
