# Correction: The association between temperature, rainfall and humidity with common climate-sensitive infectious diseases in Bangladesh

**DOI:** 10.1371/journal.pone.0232285

**Published:** 2020-04-21

**Authors:** Fazle Rabbi Chowdhury, Quazi Shihab Uddin Ibrahim, Md. Shafiqul Bari, M. M. Jahangir Alam, Susanna J. Dunachie, Alfonso J. Rodriguez-Morales, Md. Ismail Patwary

The images for Figs 4 and 5 are incorrectly switched. The image that appears as Fig 4 should be Fig 5, and the image that appears as Fig 5 should be Fig 4. The figure captions appear in the correct order.

**Fig 4:**
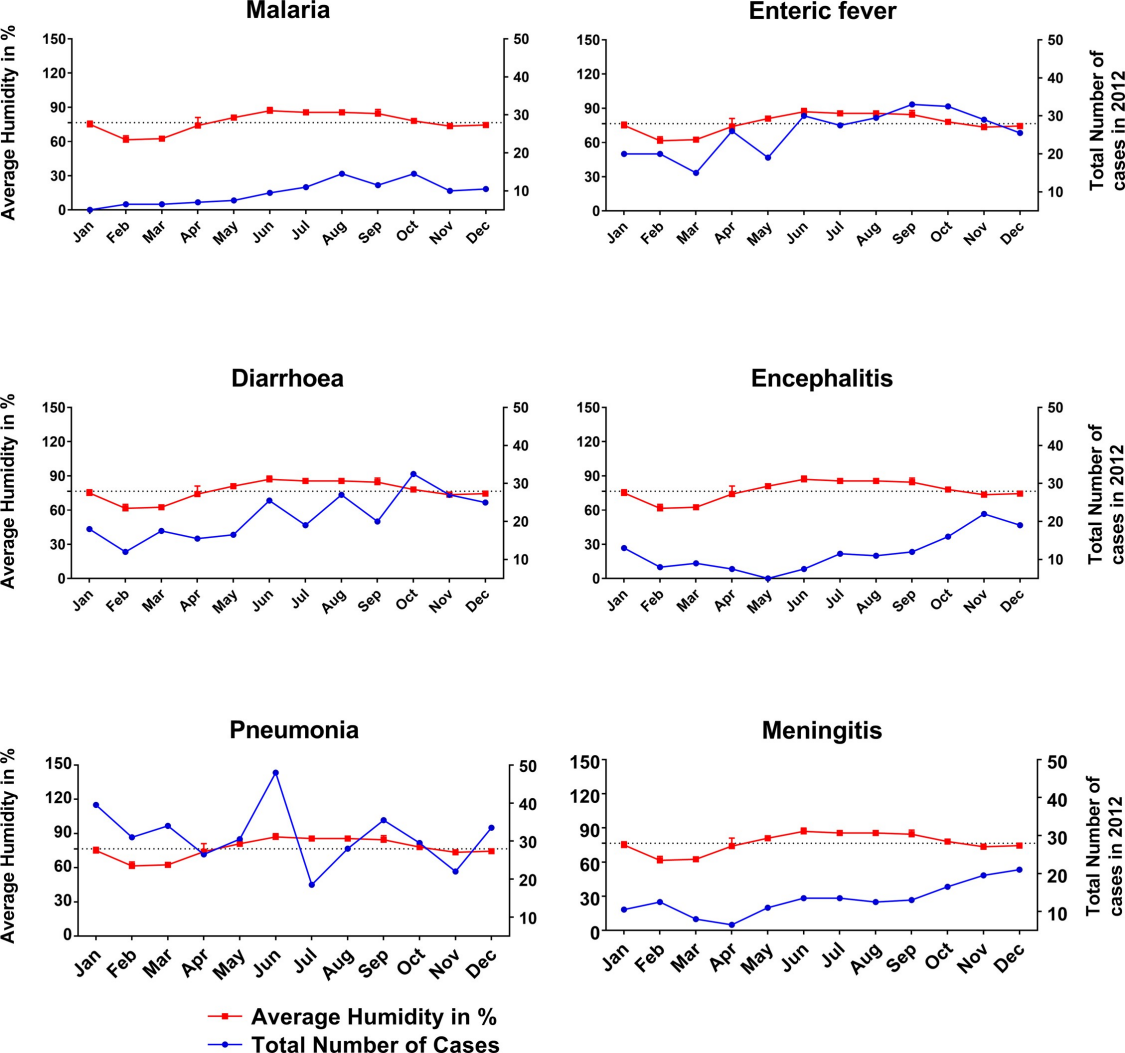
Association of disease with the average humidity for the study year 2012. Higher humidity was correlated with a higher number of cases of malaria, enteric fever and diarrhea, but inversely correlated with meningitis, encephalitis and pneumonia. Two-way ANOVA test was applied to obtain the level of significance.

**Fig 5:**
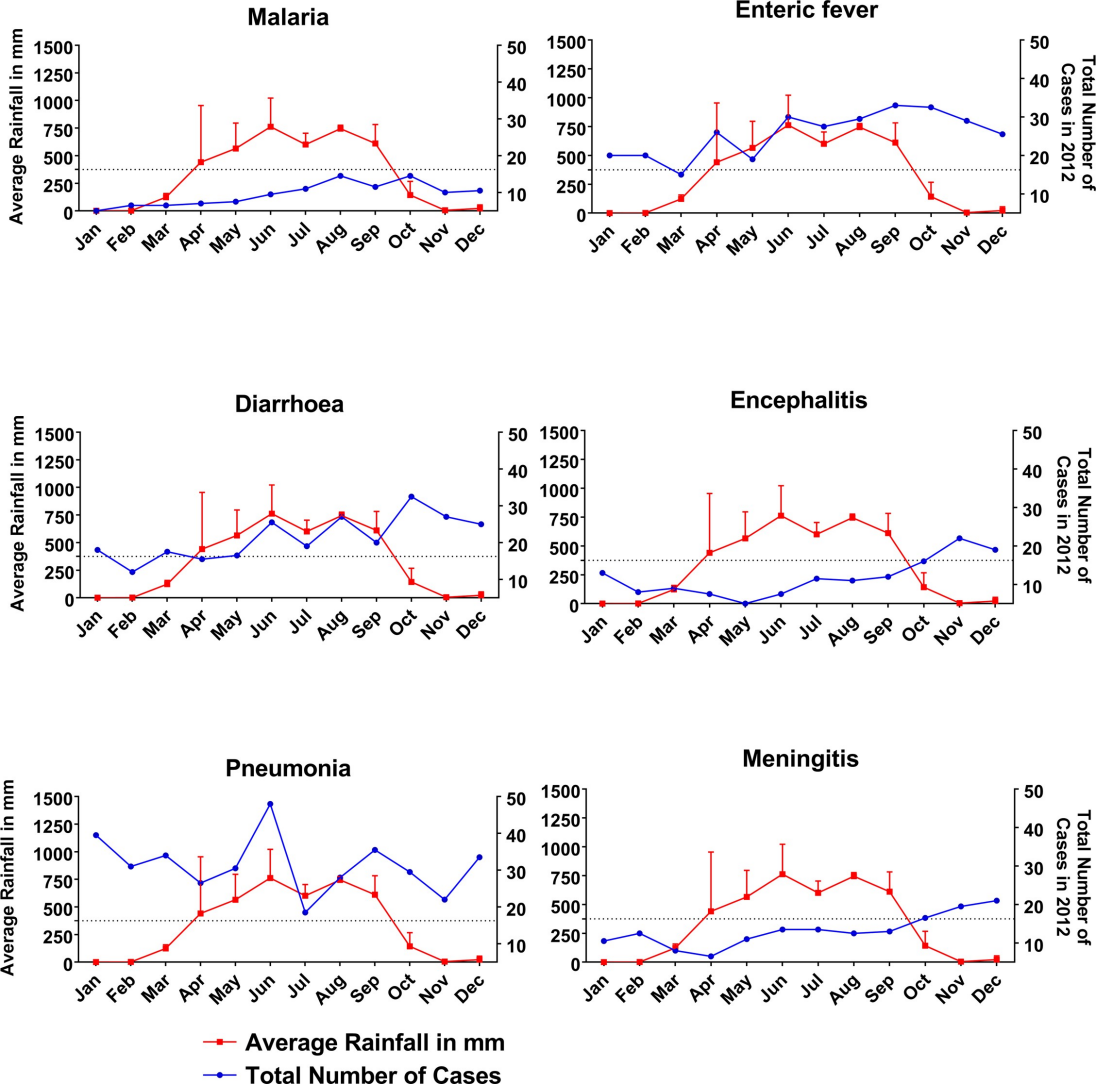
Association of disease with the average rainfall for the study year 2012. Higher incidences of encephalitis and meningitis occurred while there was low rainfall. Incidences of diarrhea, malaria, pneumonia and enteric fever increased with rainfall. Two-way ANOVA test was applied to obtain the level of significance.
